# The Role of Artificial Intelligence in the Diagnosis and Prognosis of Renal Cell Tumors

**DOI:** 10.3390/diagnostics11020206

**Published:** 2021-01-30

**Authors:** Matteo Giulietti, Monia Cecati, Berina Sabanovic, Andrea Scirè, Alessia Cimadamore, Matteo Santoni, Rodolfo Montironi, Francesco Piva

**Affiliations:** 1 Department of Specialistic Clinical & Odontostomatological Sciences, Polytechnic University of Marche, 60126 Ancona, Italy; m.giulietti@univpm.it (M.G.); m.cecati@univpm.it (M.C.); b.sabanovic@pm.univpm.it (B.S.); 2 Department of Life and Environmental Sciences, Polytechnic University of Marche, 60126 Ancona, Italy; a.a.scire@univpm.it; 3 Section of Pathological Anatomy, Polytechnic University of Marche, United Hospitals, 60126 Ancona, Italy; a.cimadamore@staff.univpm.it (A.C.); r.montironi@staff.univpm.it (R.M.); 4 Oncology Unit, Macerata Hospital, 62012 Macerata, Italy; mattymo@alice.it

**Keywords:** renal cancer, machine learning, artificial neural networks, support vector machines, random forests, NGS

## Abstract

The increasing availability of molecular data provided by next-generation sequencing (NGS) techniques is allowing improvement in the possibilities of diagnosis and prognosis in renal cancer. Reliable and accurate predictors based on selected gene panels are urgently needed for better stratification of renal cell carcinoma (RCC) patients in order to define a personalized treatment plan. Artificial intelligence (AI) algorithms are currently in development for this purpose. Here, we reviewed studies that developed predictors based on AI algorithms for diagnosis and prognosis in renal cancer and we compared them with non-AI-based predictors. Comparing study results, it emerges that the AI prediction performance is good and slightly better than non-AI-based ones. However, there have been only minor improvements in AI predictors in terms of accuracy and the area under the receiver operating curve (AUC) over the last decade and the number of genes used had little influence on these indices. Furthermore, we highlight that different studies having the same goal obtain similar performance despite the fact they use different discriminating genes. This is surprising because genes related to the diagnosis or prognosis are expected to be tumor-specific and independent of selection methods and algorithms. The performance of these predictors will be better with the improvement in the learning methods, as the number of cases increases and by using different types of input data (e.g., non-coding RNAs, proteomic and metabolic). This will allow for more precise identification, classification and staging of cancerous lesions which will be less affected by interpathologist variability.

## 1. Introduction

Renal cell carcinoma (RCC) is not a single entity but rather a heterogeneous set of tumors classified in about 40 subtypes, of which clear cell (ccRCC), papillary and chromophobe RCC account for 70%, 10–15% and 5%, respectively [1]. ccRCC is usually asymptomatic in the early stages, and about 25–30% of patients present metastasis at the time of diagnosis. Detecting ccRCC in the early stage would significantly ameliorate the prognosis, even though localized ccRCC removal by nephrectomy does not eliminate the high risk of metastatic relapse [1,2]. Therefore, also considering the increase in the number of RCC cases, development of efficient strategies for an early diagnosis and for the identification of tumors with a worse prognosis is very important.

In fact, tumor staging is not only a valuable prognostic factor, but it is also used to determine the right treatment strategy for patients and to predict the risk of metastasis development. The currently adopted prognostic factors for RCC include the TNM staging system, the four-tiered WHO/ISUP (International Society of Urological Pathology) grading system, histologic subtype, presence of the sarcomatoid component, microvascular invasion, tumor necrosis and invasion of the collecting system [3]. In case of the metastatic RCC, the most effective prognostic factors are the histological subtype and the presence of the sarcomatoid component [4]. Nomograms, models based on different prognostic factors that affect survival, have been developed to improve the prediction of patient outcomes [5,6,7]. Among these factors, or variables, we mention sex, race, marital status, smoking history, type 2 diabetes mellitus, age at diagnosis, T stage, N stage, M stage, Fuhrman nuclear grade and surgical approach.

At the same time, the use of molecular data has been explored to improve the reliability of diagnosis and prognosis in RCC. Currently, next-generation sequencing (NGS) techniques offer the opportunity to extract a multitude of new features, genomic and transcriptomic, potentially related to the phenotype. For example, gene variations, RNA expression, alternative RNA splicing events and gene fusions data from a biopsy are relatively easily obtainable [8]. However, the procedures for identification which, of the many possible variables, are indeed related to the diagnosis and/or prognosis are challenging. Thanks to bioinformatic and statistical methods, it is possible to reduce the number of variables, for example, by identifying gene groups with a correlated expression and selecting a representative gene for each group [9]. Application of these filters results in a reduced number of variables suitable for analysis by typical algorithms of machine learning. Artificial intelligence algorithms can learn the relationships among data, even if they are non-linear relationships. A simple example would be a case in which the expression of five genes is related to the diagnosis of cancer, but it is unknown what weight to attribute to each gene and what formula links the gene expression to the diagnosis. By analyzing many cases, artificial intelligence algorithms would be able to learn the relationship that links the input variables (sociological, clinical, molecular) to an output variable, such as diagnosis or prognosis or the response to treatments [10,11]. In other words, these algorithms act as classifiers that, by integrating molecular information from medical big data, will allow for the selection of specific treatments, thus making a “precision medicine”.

## 2. Machine Learning Algorithms

In this section, the main algorithms used to implement diagnostic and prognostic predictors starting from molecular variables in RCC will be summarized. However, extensive and recent reviews of the history of AI and its applications in medicine and oncology were carried out by Hamamoto et al. [12] and Hamet et al. [13]. More specific applications in urologic oncology have been carried out [14], including those based on radiological images for diagnostic and prognostic purposes in RCC [15] or those for prediction of RCC incidence over time [16,17].

Machine learning, the algorithms to implement AI, can be divided into two main types: supervised and unsupervised learning [18]. The former method is used for extracting features from input data to make predictions and address the classification and regression tasks. The classification problem is used to map input to output labels, that is, to predict discrete data, for example, distinguishing between healthy and sick. The regression problem is used to map an input to a continuous output, that is, to predict survival [19]. Unsupervised learning learns the inherent structure of data without labels being provided and the most common task is clustering. It should be noted that since no labels are used, in most cases, there is no specific way to assess model performance. Some of these algorithms include k-means clustering and principal component analysis [20]. The studies that will be examined in the next section adopted supervised learning, so now we will mention these algorithms.

The best-known artificial intelligence algorithms are artificial neural networks (ANNs), structures that mimic the neuronal topology of the human brain. They can have several artificial neurons (or nodes) organized in layers, and neurons of each layer can implement different transfer functions. All of this ensures a great flexibility in dealing with different tasks. Generally, the available cases are divided into a training set and a test set (for example, in a 70:30 ratio) but sometimes into training, validation and test sets (for example, in a 70:15:15 ratio). These algorithms perform well if the training data are numerous, representative of reality and not contradictory. Many training attempts must be made before the desired performance is achieved, and during these attempts, it may be necessary to modify the network structure, i.e., number of nodes and the type of transfer function.

While an advantage of ANNs is their ability to discover and model complex relationships among data, they present two weaknesses. Firstly, since ANNs are non-linear algorithms, training usually results in a relative minimum of the error function (between obtained and expected outputs) and not in the absolute minimum. The second is the overfitting of the data, which is a condition that occurs when the algorithm does not generalize the data profile but tries to follow them so precisely that it ends up chasing noise. Fortunately, this drawback can be easily noticed; in fact, too high performance on training data and very poor performance on test data indicate overfitting. Overfitting can also be prevented by applying methods that interrupt the learning cycles of the algorithm when the performance of the two datasets (training and test) starts to diverge [21].

Support vector machines (SVM) are also algorithms used for classification. During the learning process, these algorithms look for a hyperplane separating the two datasets (for example, healthy from sick or short from long survival). SVMs do not use all the data for the learning but rather only one datum representing the closest point between the two sets (called support vectors). Usually, these algorithms are linear; therefore, they reach the absolute minimum of the error function. Moreover, they are particularly suitable when there is a clear separation between the data groups to be classified. Instead, they have poor performance in the case of noisy data [22].

The random forests algorithm (RFs) combines the predictions of many decision trees (forests) into a single model. Each decision tree learns from a subset of elements chosen randomly from all available training data (bootstrap). Subsequently, the average of the predictions of each decision tree (bagging) is calculated, allowing obtaining the final predictions [23].

Finally, Lasso regression (least absolute shrinkage and selection operator regression) is an algorithm that performs independent variable selection (feature selection) and regularization (to reduce variance). It can select important predictors of a model [24,25].

However, there are several challenges in the application of machine learning to large amounts of data, such as genomic data. The first problem, which we have already mentioned, concerns data overfitting that occurs because the dimension of the input is much larger, at least one order of magnitude, than the sample number, and this is also known as the “large p, small n problem”. Second, the influence of each input variable on the prediction is difficult to assess because of the multiple non-linear operations. The third problem is called the “black box problem”, that is, it is not possible to understand the reason that generates the results and to predict the behavior of the system due to the complexity of the machine learning techniques. Since the European Union’s General Data Protection Regulation (GDPR) of 2018 requires the transparency of AI, it will be necessary to address the black box issue [26].

## 3. Artificial Intelligence-Based Predictors in RCC

In this review, we investigated and reported the state of art of the application of AI for diagnosis and prognosis in renal cancer using only molecular data as input, the most commonly selected genes, and we analyzed the non-AI-based predictors making a comparison between the two approaches. A Pubmed search was conducted using the keywords “artificial intelligence”, “machine learning algorithm”, “renal cell carcinoma”, “renal cancer”, “kidney cancer” and excluding the keywords “radiomic”, “imaging”, “histopathology images”, “CT-based”, “tomography” and “MRI”.

Most of published studies are focused on the prediction of prognosis and diagnosis in clear cell RCC since this is the most dominant type of renal cancer; therefore, more data are available and, consequently, the algorithms can be better trained. Only two publications, of the same authors, concern predictions on the papillary cancer type [27,28].

Usually, the RNA-seq gene expression and, in some cases, methylation data were obtained from The Cancer Genome Atlas (TGCA) resource, thus ensuring the uniformity of the starting data. Various authors used level 3 TGCA data [27,29,30], i.e., already aligned to the reference genome and quantified, but we must consider that also these processed data are dependent on the algorithm and its parameters [31]. In other papers, the transcript quantification was calculated by RSEM software [32,33].

The first phase of the analyses consists in identifying the most differentially expressed genes (DEGs) between the control and treated condition. This objective is not trivial; in fact, there are still limitations and biases that do not allow capturing all the DEGs due to the preparation of the library, the different representativeness as a function of the RNA length, the alignment of the reads and the tests for expression [34,35,36,37]. For the identification of DEGs, edgeR [38,39] and DEseq2 [40] R packages were used. Instead, differential methylation was calculated by R package limma [40] or the minfi package tool [28]. A downside in applying these methods lies in the assumption that there is no potential correlation between groups of genes. However, in biological reality, there may be gene–gene interactions that play a key role in specific conditions, whereby groups of genes could show an effect as a group, but not as single genes [41].

The second phase of the analyses consists in a reduction in the starting data (feature selection) to only the most important ones for discriminating cases, and this represents a challenge. In several studies, the feature selection was obtained through various methods, for example, using clustering algorithms, principal component analysis or random forests. In particular, as selection criteria, we start from simple methods, statistical P-values and fold changes [38]. More elaborate methods are another option, such as the “SymmetricalUncertAttributeSetEval” (of the Waikato Environment for Knowledge Analysis) and the “Fast Correlation Based Feature” algorithm [29,30], and these methods are based on performance in terms of discriminatory power (ROC) or on an SVM model [30]. It is also worth mentioning the minimum redundancy maximum relevance method [42], feature selection by the “Fast Correlation Based Feature” algorithm or joint statistical measures and logistic regression [32]. Finally, Ping et al. used the random forests algorithm for variable selection, after calculating the adjusted false discovery rate [33]. Similarly, the shrunken centroids and random forests (varSelRF) methods [27,28] and Lasso regression [39,40] were used for selecting features for predictors.

Table 1 shows a summary of the studies that used machine learning techniques for RCC diagnosis and prognosis prediction. In a study from 2014, the authors tried to discriminate ccRCC clinical tumor stages (early stages (I, II) and late stages (III, IV)) employing four different supervised machine learning algorithms (J48, naïve Bayes, sequential minimal optimization and random forest). The initial 20,534 genes from TCGA (The Cancer Genome Atlas) were reduced to 62 and their expression in 475 tumor samples was used for algorithm training [29]. The random forest based classifier reached the best performance, that is, 88.89% sensitivity, 76.84% accuracy and an auROC of 0.778.

Furthermore, in order to distinguish cancer from non-cancer samples, RNA-seq data of 537 ccRCC patients collected in TCGA were used to train a supervised learning classifier based on a support vector machine (linear kernel), and the receiver operating characteristic (ROC) curve was adopted to measure the performance of this classifier [38]. The algorithm performance seems very good, as seen from the values of sensitivity of 96.5%, specificity of 97% and the area under the receiver operating curve (AUC) of 98.7%, but unfortunately, they are referred to overall performance (training and test set). Instead, it would be interesting to evaluate the performance on test sets alone. Moreover, another weakness is that 186 genes were used, and it would be interesting to reduce the number of variables for classification.

A study published in 2015 focused on prediction of kidney cancer survival (< or ≥5 years) using TCGA RNA-seq data of 220 patients [42]. It was a complex study because the authors tested different datasets for training machine learning algorithms. In particular, both multimodal RNA-seq data (gene, exon, isoform and junction) and unimodal data (only gene, only exon, etc.) were used, and the results were compared by the area under the receiver operating curve (AUC). The support vector machine (SVM) and k-nearest neighbor (KNN) methods trained by multimodal data showed slightly better predictive accuracy (SVM_AUC = 0.6042, KNN_AUC = 0.6444) in comparison to all unimodal datasets. Unfortunately, the sample size was small, and the total accuracy of the predictions resulted limited. Similarly to Jagga et al. [29], another study used RNA-seq expression data from a slightly higher number of ccRCC cases (*n* = 523) to train SVM, random forests, SMO, naïve Bayes and J48 algorithms [30]. The SVM reached a maximum accuracy of 72.64% and an ROC of 0.81 using 64 genes on the validation dataset (RCSP-set-Weka) and similar accuracy using 38 genes (RCSP-set-Weka-Hall). However, the performance improvements were limited compared to the previously cited article. In a recent study, ccRCC patients were classified in low- and high-risk categories based on methylation data of only four genes. In fact, using the Lasso regression, classification performances assessed on the testing group by the ROC curve were 0.794, 0.752 and 0.731 for the 1-, 3- and 5-year survival rates, respectively [40]. At the same time, using only 23 genes and the SVM algorithm, an accuracy of 81.15% and an AUC of 0.86 have been achieved [32].

In 2018 and 2020, the same authors dealt with papillary renal cell carcinoma (PRCC) to discriminate between early and late stages of the disease. In particular, 104 genes were selected from gene expression profiles derived from 161 patients and used in a shrunken classifier [27]. A test on an independent RNA-seq dataset showed a maximum area under the precision recall curve (PR-AUC) of 0.81, a Matthews correlation coefficient (MCC) of 0.71 and accuracy of 88.5%. The integration of DNA methylation and gene expression data gave slightly better performance compared to the previous work [28].

A very recent study adopted different algorithms (SVM, decision tree, RF and ANN) to predict stages in ccRCC [43]. Unfortunately, large numbers of genes were used (12,897, 7251 and 6880), and the resulting performance was scarce.

We did not include two studies in Table 1 as they used learning methods only for variable selection [33,39]. Regarding these two studies, Li et al. developed a risk score model based on only 15 genes to predict the survival of patients with ccRCC who were subjected to surgery [33]. In particular, starting from gene expression data of 533 ccRCC patients, discriminating genes were selected using the random forests algorithm. The results seem in line with those obtained in the previous studies; in fact, the risk score was significantly associated with overall survival (OS) and recurrence-free survival. Moreover, the risk score for the AUC was 0.78. Meanwhile, Zhang et al. selected four differentially expressed methylation-driven genes to construct a risk score prognostic model in ccRCC [39]. For the overall survival, the AUCs for 1, 5 and 10 years were 0.734, 0.717 and 0.758, respectively.

It is difficult to compare the performance of these different prediction algorithms, since they concern different RCC subtypes and each predictor is fed with different types and numbers of genes. Furthermore, among the prognosis predictors in ccRCC, some algorithms have been trained to predict survival, and others to distinguish early from late stages, which, although they are two strongly correlated variables, are not identical. It should be taken into account that the specific parameters used by programmers to implement each specific algorithm are not known in detail. However, the best performance was achieved in the most recent study on ccRCC prognosis (AUC 0.86) which also used the fewest genes, by employing an SVM that performed better than logistic regression, multi-layer perception (MLP), random forests and naïve Bayes.

## 4. Commonly Selected Genes

We performed features comparisons to identify which selected genes were in common among studies regarding ccRCC prognosis in Table 1. Since genes can have synonymous names, we obtained their official names if these were not already adopted in the original papers, in order to obtain comparable gene lists. We identified few common genes: ATG13, HBG1 and HUS1B were the features shared among three studies, whereas CACNA1D, CASP9, CENPBD1, CTSG, EIF5B, EYA1, FABP7, FGFR3, GPR68, LINC01512, NFE2L3, RXRA, SLC22A16, SMIM3, SMLR1, TBX18, TMEM244, TNFSF4, TOB1 and UFSP2 were common between only two lists.

The HUS1B (Checkpoint protein HUS1B) gene forms a complex with Rad9 and Rad1 which are involved in response to damaged DNA, triggering cell cycle checkpoint signaling and DNA repair mechanisms [44]. CACNA1D (Voltage-dependent L-type calcium channel subunit alpha-1D) is lowly expressed in RCC [45], and the expression level of CASP9 (Caspase-9) is altered in RCC by rs12124078 SNP [46], while CTSG (Cathepsin G) inhibition enhances apoptosis in human renal carcinoma (Caki) cells [47]. The FABP7 (Fatty acid-binding protein, brain) gene is usually overexpressed in ccRCC compared to normal kidney, and its expression positively correlates with advanced clinical stage, poor survival and distant metastasis [48,49]. FGFR3°(fibroblast growth factor receptor 3) regulates cell proliferation, differentiation and apoptosis and it is frequently mutated in metastatic RCC [50] and downregulated in ccRCC [51]. High NFE2L3 (Nuclear factor erythroid 2-related factor 3) gene expression levels are associated with poor survival in ccRCC [52].

From this comparison, it emerges that different studies with the same goal, for example, prognosis prediction in ccRCC, selected different gene lists, but all their algorithms performed well. On the other hand, other studies with the specific purpose of selecting prognostic genes in ccRCC, which also used the TCGA source, obtained lists of genes that are different from each other and from the above-mentioned genes [53,54,55,56].

Despite joining all genes used by studies for ccRCC prognosis predictions, none of them were in common with the genes used for the papillary-type RCC predictions, confirming that the two cancer variants are very different at the molecular level.

## 5. Comparisons with Non-Artificial Intelligence-Based Predictors

We then analyzed the published studies which used non-AI-based predictors for diagnosis or prognosis in RCC to compare their performance with that of the predictors reported in Table 1. We selected some studies in which predictors were developed based on clustering or PCA of gene expression data. These studies, reported in Table 2, used microarray data, while studies reported in Table 1 used RNA-seq data.

Although these studies are not directly comparable to the above-mentioned ones because the AUC was not calculated, they generated lists of genes different from the above-mentioned studies but significantly associated with survival or tumor grade.

All studies demonstrated to have selected genes highly correlated to the overall survival, and one study reported a successful discrimination of patients from healthy individuals [57]. Regarding studies about ccRCC diagnosis, the genes CA9, FABP7, NDUFA4L2, PTHLH and SLC6A3 were common among the study using clustering/PCA (Table 2) and the one using learning algorithms (Table 1). Among these, FABP7 was already observed in the previously described analysis. CA9 (Carbonic anhydrase 9) and NDUFA4L2, a NADH dehydrogenase subunit, are strong candidate biomarkers for ccRCC metastasis [58,59,60]; moreover, NDUFA4L2 overexpression contributes to increase the drug resistance of ccRCC cells [61]. The overexpression of PTHLH (Parathyroid hormone-related protein) in ccRCC patients is associated with poor prognosis [62]. Further, SLC6A3 (Sodium-dependent dopamine transporter) is associated with ccRCC diagnosis and prognosis [46,63].

Regarding studies about ccRCC prognosis, we joined the genes selected from these previous studies (reported in Table 2) with those selected more recently using advanced techniques (reported in Table 1) and then we compared these two lists. The common genes were F2RL1, FABP7, GPX3, HOXC10, ITGA2, LGALS2, LGALSL, MPZL2, NNMT, RGS1, S100A4, SLPI, SPINT2, TNFAIP6, UFSP2, VCAM1 and VEGFA.

Beside FABP7, the role of GPX3 (Glutathione peroxidase 3), NNMT (Nicotinamide N-methyltransferase), S100A4 (Protein S100-A4), SPINT2 (Kunitz-type protease inhibitor 2), TNFAIP6 (Tumor necrosis factor-inducible gene 6 protein), VCAM1 (vascular cell adhesion molecule 1) and VEGFA° (vascular endothelial growth factor A) is already known. In particular, the expression of GPX3 is decreased in ccRCC [64,65]. NNMT has been suggested as a diagnostic [66,67,68] and prognostic biomarker [69]. S100A4 could be a valid prognostic marker, since it is associated with ccRCC proliferation, migration and metastasis [70,71]. SPINT2 is lowly expressed in ccRCC and may act as a tumor suppressor gene, since its knockdown induces increased invasiveness, migration and bone metastasis [72,73,74]. TNFAIP6 mRNA expression is upregulated in ccRCC [75,76,77]. In addition, the expression of its protein (TSG-6) is upregulated in inflammatory states and by growth factors [78]. VCAM1 is upregulated in ccRCC and pRCC and downregulated in chromophobe RCC and oncocytoma [79]. It is also highly predictive for survival of patients with RCC [79]. VEGFA is a growth factor involved in angiogenesis, cell migration and apoptosis. This gene is upregulated in many tumors, including RCC, and its expression is correlated with tumor stage and progression [80]. It is targeted by miR-106a-5p, but expression of this microRNA is drastically decreased in ccRCC [81].

## 6. Discussion

The use of the patient’s clinical and molecular variables is very useful for obtaining new information important for personalized therapy development. Today, we have much more molecular information available thanks to next-generation sequencing techniques and therefore more possibilities to identify the truly discriminating molecular features. There are several bioinformatic methods for the identification of discriminating variables, which subsequently will be used by the various artificial intelligence algorithms. These variables, such as transcripts, proteins or metabolites, could also represent new therapeutic targets.

Artificial intelligence systems are able to learn the relationships among data only by looking at the examples and are able to capture and reproduce non-linear relationships among the data. These algorithms are constantly being improved to ensure that they can learn better and faster and be more robust to the noise in the data. Another issue is that most machine learning algorithms are so-called “black boxes”, that is, they derive an internal model of the functioning of reality, but this cannot be explained.

These machine learning methods can also stratify patients more accurately, identifying those who present a low-stage but high-risk expression profile tumor and therefore should receive adjuvant therapies and major attention. On the other hand, patients with a high-stage but low-risk expression profile could receive less aggressive treatments under close observation. However, these objectives remain challenging, especially when considering the great molecular heterogeneity of kidney tumors.

In this study, firstly, we show that artificial intelligence algorithms yield fairly accurate predictions, even with a low number of variables (Table 1), but there is still a need to continue efforts to improve predictions. Secondly, among studies pursuing the same aim and starting from the same data (TCGA), good performance is obtained despite only a few discriminating variables being common. This may be due to the employment of different algorithms for the feature selection and the fact there are groups of genes with very similar expression profiles for which different algorithms choose different genes to represent the same class. Third, the comparison between AI- and non-AI-based predictors was not possible since different parameters are used to describe performance. For the same reason, the comparison with nomograms is not possible when the C-index is provided [5,86,87] but only when the AUC is present. In this case, a nomogram reached an AUC of 0.813 and 0.799 for the 3-year and 5-year survival, respectively [7]. Similar performance (0.801 AUC) is obtained by integrating expression data in predicting a high ISUP (International Society of Urological Pathology) grade of ccRCC [88]. These results, obtained with very simple and transparent systems, are similar to or slightly lower than those of AI systems.

In colon and breast cancers, AI predictors reached an accuracy of 0.767 and 0.807, respectively, for disease recurrence [89]. Better results were obtained in the prediction of survival at 1 year and 5 years in esophageal carcinoma (0.883 and 0.884 AUC) [90]. Therefore, in other cancers, the results of the predictions are similar to those obtained for RCC.

To increase the accuracy of predictions in prognosis, data on mutations have been integrated with those of gene expression [91,92,93]. However, it is difficult to train an expert system to consider the mutation load of a sample since the effect of a mutation depends on the function of the gene and its position along the gene [94]. Unfortunately, since there is no such detailed information, all mutations are grouped together, and this diminishes the predictive power of expert systems.

In the future, thanks to the greater availability of data in TCGA, it will be possible to realize gender-, ethnic- and RCC variant-specific predictors.

## 7. Conclusions

AI-based predictors are powerful tools that can be continuously trained as new data become available. In this review, we summarized recent studies that adopted these predictors for diagnosis and prognosis in RCC. We show the good performances obtained so far, but also the need for improvement in order to achieve real clinical usefulness.

## Figures and Tables

**Table 1:** The table collects the studies that used machine learning techniques to predict diagnosis and prognosis.

Aim	Technique and Data	Results	Reference
ccRCC stages (I and II vs. III and IV)	J48, naïve Bayes, sequential minimal optimization and random forest on RNA-seq data from TCGA	62 genes selected. Random forest was the best predictor: 88.89% sensitivity, 76.84% accuracy and auROC of 0.778	Jagga et al., 2014 [29]
ccRCC vs. normal	SVM on RNA-seq data from TCGA	186 selected genes, overall sensitivity 96.5%; overall specificity 97%; overall AUC 98.7%	Yang et al., 2014 [38]
ccRCC survival (< or ≥5 years)	SVM and KNN learning on RNA-seq data from TCGA	SVM (AUC 0.6042; total accuracy 0.6111) KNN (AUC 0.6444; total accuracy 0.6481)	Schwartzi et al., 2015 [42]
ccRCC stages (I and II vs. III and IV)	SVM, random forests, SMO, naïve Bayes, J48 on RNA-seq data from TCGA	64 and 38 genes selected. SVM was the best predictor: sensitivity 73.44%, specificity 71.43%, accuracy 72.64%, 0.81 ROC (on validation data)	Bhalla et al., 2017 [30]
Papillary RCC stages	KNN, SVM, naïve Bayes, random forests, shrunken centroid	104 selected genes. Shrunken was the best predictor: PR-AUC 0.81, MCC 0.71, accuracy 88.5% (in an independent dataset)	Singh et al., 2018 [27]
ccRCC survival	Lasso regression on TCGA data	4 gene methylation data. According to ROC curve: 1-year survival rates 0.794, 3-year 0.752, 5-year 0.731	Tang et al., 2020 [40]
ccRCC stages (I, II and III, IV)	SVM, logistic regression, MLP, random forests and naïve Bayes on TCGA data	23 genes selected. SVM was the best predictor: accuracy 81.15%, AUC 0.86 (in a testing set)	Li et al., 2020 [32]
Papillary RCC stages	Random forests, naïve Bayes, linear-SVM, KNN, shrunken centroid, group Lasso, BEMKL on TCGA data	DNA methylation in addition to previously selected [27] 104 features. Random forests and group Lasso (for both MCC 0.77, PR-AUC 0.79, accuracy 90.4)	Singh et al., 2020 [28]

PCA: principal component analysis; AUC: area under the receiver operating curve; SVM: support vector machine; MLP: multi-layer perception; RF: random forest; KNN: k-nearest neighbor; auROC: area under the receiver operating characteristic curve; PR-AUC: maximum area under the precision recall curve; MCC: Matthews correlation coefficient; BEMKL: Bayesian efficient multiple kernel learning.

**Table 2:** The table collects the studies that used different techniques than machine learning to predict diagnosis and prognosis.

Aim	Technique and Data	Results	Reference
ccRCC survival at 5 years	Clustering on cDNA microarray (29 patients)	40 genes correlated with survival (Kaplan–Meier, *p* < 0.0001) and histological grade.	Takahashi et al., 2001 [82]
metastatic ccRCC subgroups	Clustering on cDNA microarray (58 patients)	45 genes distinguishing groups for overall survival (*p* = 0.001)	Vasselli et al., 2003 [83]
ccRCC survival after surgery	Clustering and supervised PCA on cDNA microarray (177 patients)	259 genes correlated with survival (*p* < 0.001 by the log-rank test on test set)	Zhao et al., 2006 [84]
		Top 4 genes correlated with survival (*p* = 0.02)	
ccRCC vs. normal	Clustering and PCA on cDNA microarray (16 patients)	21 genes over expressed in ccRCC compared to normal tissues	Skubitz et al., 2006 [57]
ccRCC subgroups		Fewer genes distinguishing 2 ccRCC subgroups likely related to pathologic grade	
ccRCC survival	PCA and clustering and logical analysis on cDNA microarray (48 patients)	110 genes associated with tumor stage (*p* = 0.009) and grade (*p* = 0.0007) and survival (median survival of 8.6 vs. 2.0 years, *p* = 0.002)	Brannon et al., 2010 [85]
